# Biofortification of Kidney Bean (*Phaseolus vulgaris* L.) Crops Applying Zinc Sulfate and Ferric Sulfate: Pilot Crop in Colombia

**DOI:** 10.3390/molecules28052004

**Published:** 2023-02-21

**Authors:** Camilo Andrés Guerrero-Martin, Angie Tatiana Ortega-Ramírez, Óscar Silva-Marrufo, Braian David Casallas-Martín, Natalia Cortés-Salazar, Raúl Salinas-Silva, Stefanny Camacho-Galindo, Fernando Antonio Da Silva Fernandes, Laura Estefanía Guerrero-Martin, Pedro Paulo de Freitas, Emanuele D. V. Duarte

**Affiliations:** 1 LOTEP—Laboratório de Operações e Tecnologias Energéticas Aplicadas na Indústria do Petróleo, Faculty of Petroleum Engineering, Federal University of Pará, Salinópolis 68721-000, PA, Brazil; 2 Department of Engineering, Campus Salinópolis, Federal University of Pará, Salinópolis 68721-000, PA, Brazil; 3 LEEPER—Laboratório de Ensino de Engenharia de Poco e Reservatório, Faculty of Petroleum Engineering, Federal University of Pará, Salinópolis 66075-110, PA, Brazil; 4 Management, Environment and Sustainability Research Group, Chemical and Environmental Engineering Department, Universidad de América, Bogotá 110311, Colombia; 5 Departament of Engineering, Tecnológico Nacional de Mexico, Instituto Tecnológico del Valle del Guadiana, Carretera 34371, Durango, Mexico; 6 Faculty of Agronomy, Tecnologica University of Rodeo, Rodeo 35760, Durango, Mexico; 7 Fundación de Educación Superior San José, Bogotá 110311, Colombia; 8 Department of Biosystems Engineering, University of São Paulo, Pirassununga 13635-900, SP, Brazil

**Keywords:** agriculture, beans, biofortification, zinc, iron

## Abstract

Agriculture is one of the economic activities with the most potential in Colombia, given its climatic and geographical conditions. Bean cultivation is classified as climbing, which grows in a branched way, and bushy, whose growth occurs up to 70 cm. The objective of this research was to study zinc and iron sulfates in different concentrations as fertilizers capable of increasing the nutritional value of kidney beans (*Phaseolus vulgaris* L.), whose strategy is known as biofortification, and thus determine the most effective sulfate. The methodology details the sulfate formulations, their preparation, the application of additives, sampling and quantification methods of total iron, total zinc, °Brix, carotenoids, chlorophylls a, b, and antioxidant capacity using the DPPH (2,2-diphenyl-1-picrylhydrazyl) method in leaves and pods. As for the results, it was found that biofortification with iron sulfate and zinc sulfate is a strategy that favors the country’s economy and human health, because it allows the increase of minerals, antioxidant capacity and total soluble solids.

## 1. Introduction

Agriculture significantly influences the economy of the rural areas in Colombia; its contribution to the country is related to sustainable growth, as it allows poverty reduction by increasing the labor supply, and ensuring the availability of food and the progress of the economy, which suggests the commitment of this sector to the fulfillment of the 17 Sustainable Development Goals (SDGs) [1,2]. According to the above, the National Administrative Department of Statistics—DANE—reported that the agricultural and agro-industrial sector showed the economic activity with the highest growth during 2020. For its part, the World Bank stated that Colombian agriculture, in the 2011–2015 period, contributed 6.3% to gross domestic product (GDP) [1,2,3,4].

As a country located in an intertropical zone, Colombia has benefited from varied climates, soils, ecosystems, and other natural resources that favor the growth of plant species, representing an advantage for agriculture [5,6,7]. The FAO shows that the country can expand its agricultural territory (without affecting natural reserves), ranking seventh in Latin America’s cultivable extension, which implies an economic advance and, consequently, a change to supply, highlighting that the territory only uses 4.8 million hectares of the 22 million arable hectares [5,7,8].

Based on the above, one of the most important crops in Colombia is beans, which are found in different regions of the country, specifically in the Andean region in the departments of Huila, as the leading producer, followed by Antioquia, Boyacá, Santander, and Tolima. However, Bolívar and Nariño are also part of the top-producing departments [9,10,11]. Beans are one of the most consumed foods in Colombian households, with a high nutritional value of proteins and minerals, to the extent that in 2018 they yielded between 3 and 4 kg per capita. Due to this high demand, more production is required, and, therefore, the employability and income generated increase [12].

There are two bean classes: the first corresponds to the climbing bean, which grows in a branched form and covers 65% of the national production, and the second involves the bush bean that does not grow more than 70 cm, and represents 35%; these figures explain consumers’ preference for the large-grain bean, typical of the first classification mentioned [9,12]. Regular bean crops in Colombia correspond to “Froilán”, white charger, red charger, Calima, and kidney bean (*Phaseolus vulgaris* L.), the latter being the relevant ones for this research [9,13]. Meanwhile, for 2020, the area planted with beans in Colombia was 35,200 ha, producing 68,487 tons and a yield of 1.25 tons ha-1 [13,14].

The agronomic management implemented for the bush bean occurs in conditions close to 12–27 °C, and to begin with, includes preparing the soil to ensure a higher yield and plant blossoming. Soil correction and fertilization must be carried out by analyzing the physicochemical properties, which must include loam content with a pH between 5.5 and 6.5 to quantify the nutrients, since the organic-matter level needs to be significant and water-retention efficient. Next the planting system and plant-density determination; the crop cycle is short, and the distance between rows is 60 cm and the plants are 7 cm apart [9,13,15].

The farmer must monitor weeds in two ways: chemical control, which principally uses glyphosate as a pre-sowing herbicide, and manual control, deployed after sowing and before flowering. At this moment, it is convenient to include disease and pest control, highlighting the fact that foliage pests (chrysomelids and chewing worms) and biting pests (whitefly and aphids), along with anthracnose diseases and the common mosaic virus, are the most frequent in these crops [9,15,16]. The last stage of agronomic management refers to the harvesting, where the best pods are selected in terms of size, shape, number of seeds, and health; the moisture is approximately 20% and the pods are physiologically mature [13,14,15,16].

The growth of beans comprises the vegetative stage, which includes germination, emergence, primary leaves and, the first and third trifoliate leaf, and the reproductive stage, which has pre-flowering, flowering, pod formation, pod filling, and maturation or physiological maturity [9,10,11,15,16,17]. Nitrogen, phosphate, and potassium fertilization with additives such as ammonium sulfate, ammonium nitrate, urea, potassium sulfate, and organic matter, among other fertilizers, provide nitrogen, potassium, and calcium, and to a lesser extent, sulfur, magnesium, and phosphorus to the pod and stem of the bean, according to the mineral-contribution requirements [18]. However, a strategy called biofortification is implemented to increase the nutritional value of beans, using additives that raise the levels of vitamins (vitamin A) and minerals (zinc, iron, and iodine) from bean to bean, up to the concentrations of micronutrients desired by consumers and farmers [19].

Given the efficacy, profitability, and feasibility demonstrated by biofortification, it has become a response to public-health problems such as reduced mental capacity and abnormal thyroid growth due to iodine deficiency; anemia, motor disability, cognitive impairment, and complications in pregnant women caused by iron deficiency; visual impairment, diarrhea and measles in early childhood caused by vitamin A deficiency; and weakened immune system and cellular atrophy, due to zinc deficiency [18,19]. For example, 1.9 million people worldwide suffer from anemia and one-third from diseases associated with zinc deficiency. Likewise, in Colombia, 27.5% of children between 3 and 5 years old, 17.9% of pregnant women, and 7.6% of women between 10 and 54 years of age suffer from anemia [20].

Thus, this research focuses on implementing agronomic biofortification as a strategy to increase the nutritional value of red kidney beans (*Phaseolus vulgaris* L.) through the chemical additives iron and zinc sulfate in different concentrations, using foliar application to the plants. In this way, zinc sulfate is used as a fertilizer to increase the presence of the mineral in the crops, either by foliar application or by irrigation systems; likewise, iron sulfate is used as a corrector of the insufficiency of this nutrient in the soil, lowering the pH of the micronutrients it contains and releasing them [21,22,23].

## 2. Results

### 2.1. Determination of Iron and Zinc in Bean Leaves and Pods

Table 1 denotes the results of the final content of iron and zinc both in the pod and in the leaf accumulation, after applying the iron sulfate in the mentioned concentrations. The highest quantity of iron content in the pod was 0.050 µmol dm^−3^ of sulfate in almost all the variables, both in the pod and the fruit, with a concentration of 1145 mg kg^−1^; this also occurred with the same concentration of sulfate, but in the leaf, as there was 171 mg kg^−1^ of iron content. However, in the case of zinc, the percentages accumulated in the leaf and captured by the pod were lower when adding iron by foliar means.

Table 2 shows the results of the final accumulation of iron and zinc in the leaf and pod after applying zinc sulfate at the above-mentioned concentrations. The highest zinc content in the pod was 0.200 µmol dm^−3^ of sulfate (according to the standard deviation), with a concentration of 11 mg kg^−1^. Likewise, the highest content of this mineral in the leaf was from 22 mg kg^−1^ to 0.200 µmol dm^−3^. However, the iron content in both cases is more significant; hence, it will be discussed in the next section.

Both tables show an initial concentration of 0 µmol dm^−3^ (analytical blank), which is a critical comparison point for the present research when using the other concentrations.

### 2.2. Chlorophyll, Carotenoids and °Brix 

Chlorophyll is a photosynthetic pigment produced in the chloroplast, and a color provider to the plant. It also allows photosynthesis to be conducted satisfactorily, absorbing sunlight. There are several types of chlorophyll, although this article addresses types a and b, which differ in their wavelength and molecular structure. Secondly, carotenoids are organic compounds responsible for the color of fruits and vegetables and for ultraviolet protection during photosynthesis, since they scavenge oxygen and free radicals [24,25]. In continuation, Table 3 shows the average of the variables total soluble solids (TSS), chlorophyll a, chlorophyll b and carotenoids, in the parts of the bean plant: bean, pod and leaf. It is important to note that Table 3 shows the treatment with iron sulfate.

According to Table 3, in the case of iron sulfate as a fertilizer, it was observed that the highest concentration of chlorophyll a and b was 0.730 mg dm^−1^ DW, and for carotenoids it was 0.013 mg dm^−1^ DW. In this case, the quantity remained almost constant, except for the second treatment, at a concentration of 0.025 µmol dm^−3^ of the additive, which implies that the remaining treatments are statistically alike. At the same time, Table 4 shows the same study variables as Table 3, but with the treatment with zinc sulfate.

Table 4 enhances the fact that the highest concentration of chlorophyll a and b was 0.858 mg dm^−1^ DW in both cases, using zinc sulfate as a fertilizer. Nevertheless, on this occasion, the total concentration of carotenoids was constant, so there were no significant differences in treatments, with *p* ≤ 0.05. In both situations, we obtained the results within 22 days.

However, the content of total soluble solids (TSS) expressed in Table 3 and Table 4 indicates that in both cases (iron and zinc sulfate), the Brix degrees in the different treatments were higher, in respect of the concentration of the control. It suggests that the additives had a positive effect, increasing the carbohydrate content in the bean grain. In the case of iron, focusing on the results of the pod at concentrations of 0.025, 0.050, 0.100, and 0.200 µmol dm^−3^, the values are statistically the same; however, these results come close to the control value, concluding that the additive did not exceed expectations of nutritional increase for the pod.

### 2.3. Antioxidant Activity

In the case of the kidney bean (*Phaseolus vulgaris* L.), iron sulfate favored its antioxidant activity concerning zinc sulfate, since the TEAC-Trolox Equivalent-Antioxidant-Capacity value for the first additive mentioned was 12.36 mg eq. Trolox/100 g, while for the second it was 10.12 mg eq. Trolox/100. 

## 3. Materials and Methods

### 3.1. Experimental Location

The research was carried out in the municipality of Chocontá, Cundinamarca (Colombia), whose location is in the Colombian Cundinamarca plateau at the coordinates 5°8′45″ North and 73°41′8″ West, latitude 5.14583 and longitude −73.6855. Its average annual temperature is 13 °C, and it has frequent rainfall, so it offers favorable climatic conditions for the growth of plants such as red beans (*Phaseolus vulgaris* L.) [26]. The bean growth place is a rural area where the predominant economic activity is agriculture, and biofortification is being prioritized as a scientific alternative to increase nutrients in food. The experiment was established in the aforementioned field; the samples were collected by treatments and repetitions for each plant, transported, and a large part of the analysis was carried out in the general biotechnology laboratory belonging to the Fundación Universidad de America, located in Bogota, D.C. Colombia. The analyses of the antioxidant capacity of the plant samples were carried out in a laboratory outside the University.

### 3.2. Formulation of Iron-Sulfate and Zinc-Sulfate Additives in Bean Leaves and Pods

The preparation of the iron-sulfate (Roda Químicos S.A.S, Bogotá D.C., Colombia) and zinc-sulfate (Roda Químicos S.A.S, Bogotá D.C., Colombia) additives is defined from the formulation of each of them, as follows:

In Table 5, the amounts of each substance (e. g., 0.00379770 g of iron sulfate and 0.00404 g of zinc sulfate, respectively, for preparing each fertilizer in the second scenario) were taken, pre-weighed on an analytical balance, and added by means of an atomizer containing 1 dm^−3^ of water from a local stream; (therefore, the dosage was 0.25 dm^3^ per plant). This was done so that the four fertilizers were prepared with different concentrations of each additive, and comparing them would give better results in bean biofortification.

### 3.3. Additive-Application Method and Furrow Distribution

In addition, bean-crop additives were applied in five treatments using sulfate concentration; i.e., four plants were subjected to the foliar application of the fertilizer and, likewise, zinc sulfate. Then, each of the additives was added to two different rows with 20 plants on the surface, and to the roots of the leaves. Since the objective was the preparation of two different additives, iron and zinc, and to find out the benefit of each one individually on the nutritional increase of the kidney bean (*Phaseolus vulgaris* L.), it was a two factor experiment, with Fe and Zn, in which the variable was the concentration of each nutrient during the time, taking into account the fact that each experiment had five series. Thus, Table 6 and Table 7 indicate the concentrations of zinc sulfate and iron sulfate representing each treatment, respectively.

Consequently, the first series of plants on each application of sulfate (T_1_, T_2_, T_3_ and T_4_) served as the analytical target of each furrow, so the additives were not applied at any concentration. Then, the following four plants were placed, applying the first concentration of each sulfate: plants number 5–8 of the zinc-sulfate furrow were irrigated with this fertilizer at a concentration of 0.00695025 g dm^−3^ and 0.0071885 g dm^−3^. The same procedure occurred with the iron-sulfate furrow, at a concentration of 0.0071885 g dm^−3^ and 0.0695025 g dm^−3^. This fertilizer irrigation was performed on each plant at a frequency of 8 days until completing the study, i.e., the application was made three times during 24 days. Samples were collected approximately 1 month after adding the sulfates. For each of the treatments, about 200 g of green and intact leaves and pods were collected. The samples were used for different studies to provide results to discuss later in this article. 

### 3.4. Quantification of Iron and Zinc in Kidney Beans (Phaseolus vulgaris L.) 

In the first step, the content of iron and zinc in the pods and leaves of each treatment was determined using an XRF spectrometer NITON XL3t (Thermo Scientific, Waltham, MA, USA). This detects heavy metals in soil, water, and plant material, which need to be standardized, according to the American Wood Preservers Association, AWPA. The spectral analysis of the X-rays re-emitted by fluorescence is the analytical technique that allows this equipment to obtain the characteristics of the different chemical elements in the sample. Thus, the analysis of the relative intensities for each sampling allows for concentrations of elements [26,27]. It should be noted that the equipment used to detect heavy metals, as well as essential and non-essential nutrients, detects them directly towards the plant sample due to the X-ray fluorescence it contains, and it is not necessary to prepare previous solutions to quantify some nutrients, since it is portable equipment and easy to use.

### 3.5. Determination of Chlorophyll a and b, Carotenoids and °Brix 

In this study, the quantification of chlorophylls and carotenoids was determined, based on the Wellburn methodology [28], with some modifications: 60 mg of lyophilized tissue were added to a test tube, to which 10 mL of 98% methanol were subsequently added according to the cited authors, 98% methanol was used, to allow incubation at room temperature (20 °C), with no light, for 24 h. Finally, the UV spectrophotometer Genesys30sV (Thermo Scientific, Waltham, MA, USA) read the absorbances at wavelengths of 666, 653, and 470 nm. All concentrations of photosynthetic pigments are expressed in milligrams per gram of dry weight (mg dm^−1^ DW) through the Equations (1)–(3):

(1) Chl a=[(A666)−(A653)]

(2) Chl b=[(A653)−(A666)]

(3) $$Carotenoids=\frac{\left[\left(A470\right)-\left(Chl\;a\right)-\left(Chl\;b\right)\right]}{221}$$

The °Brix quantifies the concentration of sugars in the fruit. More specifically, these sugars can be associated with total soluble solids (TSSs): minerals, pigments, acids, and sugars, among others [29]. The TSSs were determined through the Balaguera et al., methodology [30], consisting of two pods and three seeds taken from each treatment, of which the plant material was subjected to maceration in a porcelain mortar using methanol at 3% one day after the harvest, to obtain the extract that would later be stored in a digital refractometer 300034 (Sper Scientific, Phoenix, AZ, USA). The figures from s the measuring instrument are given in °Brix [31].

### 3.6. DPPH (2,2-diphenyl-1-picrylhydrazyl) Determination 

The extracts obtained following the methodology of Balaguera [30] were used for the DPPH assay. The sample antioxidant capacity established whether the plant managed to delay oxidative degradation by the action of additives (chain-terminating antioxidants; sulfates, in this case), and therefore increased the ability to react with free radicals [29,30,31]. Therefore, the antioxidant analysis of the sample was carried out in the AOXLAB SAS laboratory, whose methodology is based on UV-visible method using a UV spectrophotometer Multiskan SkyHigh Microplate (TermoFisher Scientific, Waltham, MA, USA) using 0.01 mL of the sampling and 0.99 mL of the DPPH methanolic solution (20 mg/L). As an analytical blank, the same amount of DPPH and 0.01 mL of the sample solvent (DMSO) were added, followed by the absorbance reading of the sample at a wavelength of 517 nm, after 30 min of the previous reaction at room temperature had elapsed. Radical inhibition is calculated with a standard curve, elaborated after measuring various concentrations of TROLOX antioxidant, which expresses its results in TEAC or Trolox-Equivalent-Antioxidant-Capacity values.

### 3.7. Statistical Analysis

The results were analyzed by variance and comparison of means with Tukey’s test (*p* ≤ 0.05), using the statistical software SPSS version 15^®^ [32].

## 4. Discussion

### 4.1. Composition of Iron and Zinc in Bean Leaves and Pods

The results of Table 1 and Table 2 shows that with a concentration of 0.025 µmol dm^−3^ of iron sulfate, the leaf and pod change their iron composition; however, the results do not necessarily imply a radical increase, due to the foliar application of the additive. Thus, it is uncertain to state that at higher concentrations there is an improved bean; even so, it is certain that at medium concentrations, both the pod and the leaf will increase their micronutrient composition, or rather, their nutritional value. Accordingly, while minimal zinc uptake was experienced in the leaves—being almost constant—, iron uptakes were mostly significant compared to Guillén-Molina et al. [33], who added low doses of Fe without Zn (36.8%,), increasing the content of Fe (12.6%,), and Mn (26.5%), especially in the application of zinc sulfate to the leaves. In this study, the dose of 0.025 µmol dm^−3^ combined with Fe and Zn increased Fe and stabilized Zn.

In the case of zinc sulfate, the increase in Zn is not relevant compared to Fe; that is the case of the concentration of 0.100 µmol dm^−3^ of this additive, in which the iron composition of the pod and leaf was 91 mg kg^−1^ and 55 mg kg^−1^, respectively. However, the zinc composition of the pod and leaf at the same concentration was 8 mg kg^−1^ and 7 mg kg^−1^, respectively, because the plant naturally possesses a higher presence of iron, in contrasted to zinc. The results allow us to “visualize” that zinc as sulfate does not represent an advantage to the plant by fertilizing, as it does in chelate form, since Preciado-Rangel et al. [34] compared zinc with zinc sulfate, finding that at a concentration of 0.025 µmol dm^−3^, zinc chelate improved bean quality and, therefore, consumer health.

### 4.2. Composition of Chlorophyll, Carotenoids and °Brix

For their part, Quintana-Blanco et al. [35], point out that the chlorophyll content decreased as the concentration of the additive increased, that is, at higher doses of additive; however, in the present investigation the results show that they are random, or they seem to be random. In other words, the chlorophyll content is independent of the sulfate concentration: it decreases in an inversely proportional way to the additive concentration. This statement is ratified in Table 3, where the maximum chlorophyll a and b occurs in treatment 1, followed by treatments 2, 4, and 5, until reaching the minimum in treatment 3; and in Table 4, when the maximum chlorophyll a and b occurs in treatment 5, then in treatments 2, 1, and 4, until reaching the minimum of this pigment in treatment 3, which leads to the fact that at 0.050 µmol dm^−3^ for zinc sulfate and iron sulfate, the lowest contents of chlorophyll a and b are obtained in the leaves; hence, it is an unfavorable treatment for photosynthetic development. These effects have been observed in various crops, such as beans; as reported by Zewail [36], the present results did not show a convincing response; in the case of total carotenoids, no difference was observed, while chlorophylls a and b had minimal uptakes with the contributions of iron and zinc by the foliar route in bean plants; that is, it is not a favorable treatment for photosynthetic development [37,38,39]. 

By increasing the mineral concentration in the leaves of the bean plant of kidney variety (*Phaseolus vulgaris* L.), we expected a proportional increase in the total chlorophylls and total carotenoids as a sought physiological response indicated by García-Cruz et al. [40], in different bean crops. However, in contrast to this version, a different result was obtained, where the physiological response of the plant was not favored by the presence of both minerals, since the total carotenoids described by Corzo-Piñeros et al. [41] did not vary from one treatment to another, and the contents of chlorophyll a and b did not experience a significant increase; accordingly, the contributions of iron and zinc through foliar application to bean plants, were minimal.

### 4.3. Antioxidant Capacity of Kidney Beans (Phaseolus vulgaris L.)

Antioxidants are crucial for the body, and create a barrier against reactive oxygen species [34,35,36], so frequently consumed foods must have significant antioxidant activity. For this reason, iron has a natural capacity to neutralize free radicals such as DPPH through a redox reaction after interacting with them, thus efficiently preventing damage to the body. It should be noted that the present studies were carried out for the purpose of showing which concentration is suitable for obtaining beans with a high content of iron and zinc.

## 5. Conclusions

Biofortification with iron and zinc sulfate at concentrations of 0.025, 0.050 and 0.100 µmol dm^−3^ increased the content of these minerals, the antioxidant capacity, and the carbohydrate ratio, both in the leaf and in the pod of the bean of the kidney variety (*Phaseolus vulgaris* L.). The results propose an improved alternative for human health and the country’s economy that can be a scalable strategy for different types of crops. In both additives, the iron composition was higher since, from the beginning, this component prevailed concerning zinc; in addition, it provided more antioxidant activity in the plant substrate by having more interaction with the DPPH free radical.

It should be noted that the response variables such as iron and zinc content, and total soluble-solids content do not show a constant decreasing or increasing behavior, implying that the process variable of initial sulfate concentration does not generate dependence on these response variables, being purely random. 

We suggest implementing a cultivation strategy that involves the addition of sulfates when sowing the kidney bean (*Phaseolus vulgaris* L.), to have a broad spectrum of results and achieve a better comparison from start to finish. Although the fertilizer-application method was foliar, the possibility of applying the same root or fertigation route should be evaluated to conclude which is the most effective. Due to the analysis of the samples corresponding to the plants and pods collected on the last day, we find it favorable to collect these plant species, the kidney bean, twice before ending the research, for example, every five days.

## Figures and Tables

**Table 1:** Average micronutrient content of Zn and Fe in foliar tissue and bean pod, due to iron application in different concentrations.

Treatments of Iron Sulfate (µmol dm^−3^)	Zn **	Fe *	Zn **	Fe *
	Leaf		Pod	
	mg kg^−1^		mg kg^−1^	
0.000	7 ± *** 3 a	71 ± *** 13 b	6 ± *** 4 ab	42 ± *** 15 b
0.025	9 ± *** 3 a	69 ± *** 13 b	9 ± *** 4 ab	43 ± *** 15 b
0.050	0 b	171 ± *** 20 a	19 ± *** 6 a	1145 ± *** 44 a
0.100	11 ± *** 3 a	102 ± *** 15 ab	6 ± *** 4 ab	63 ± *** 16 b
0.200	0 b	77 ± *** 16 ab	6 ± *** 4 ab	94 ± *** 18 b

* Fe: iron; Zn: zinc. ** Values with same letters within a column are statistically similar (Tukey; *p* ≤ 0.05). *** ± = Standard deviation.

**Table 2:** Average micronutrient content of Zn and Fe in foliar tissue and bean pod, due to zinc application in different concentrations.

Treatments of Zinc Sulfate (µmol dm^−3^)	Zn **	Fe *	Zn **	Fe *
	Leaf		Pod	
	mg kg^−1^		mg kg^−1^	
0.000	9 ± *** 4 ab	16 ± *** 16 ab	8 ± *** 3 a	39 ± *** 12 ab
0.025	14 ± *** 5 a	105 ± *** 20 a	0 b	25 ± *** 13 ab
0.050	9 ± *** 5 a	92 ± *** 19 a	0 b	32 ± *** 15 a
0.100	8 ± *** 4 ab	91 ± *** 17 ab	7 ± *** 4 a	55 ± *** 15 a
0.200	22 ± *** 4 ab	88 ± *** 17 ab	11 ± *** 4 a	38 ± *** 14 a

* Fe: iron; Zn: zinc. ** Values with same letters within a column are statistically similar (Tukey; *p* ≤ 0.05). *** ± = Standard deviation.

**Table 3:** Average of the variables total soluble solids (TSS), chlorophyll *a*, chlorophyll *b*, and carotenoids.

Treatments of Iron Sulfate (µmol dm^−3^)	TSS * °Brix		Chlorophyll *a* (mg dm^−1^ DW **)	Chlorophyll *b* (mg dm^−1^ DW **)	Total Carotenoids (mg dm^−1^ DW **)
	Bean	Pod	Leaf		
0.000	0.53 c	5.7 b	0.730 a	0.073 a	0.013 a
0.025	1.80 c	5.8 b	0.660 a	0.066 a	0.012 b
0.050	5.10 b	6.7 a	0.190 b	0.019 b	0.013 a
0.100	6.50 b	6.7 a	0.470 ab	0.047 ab	0.013 a
0.200	9.20 a	5.5 b	0.220 b	0.022 b	0.013 a

* TSS: °Brix; DW: Dry Weight. ** Values with same letters within a column are statistically similar (Tukey; *p* ≤ 0.05). Note: chlorophyll a: results with minus are with letter b.

**Table 4:** Average of the variables total soluble solids (TSS), chlorophyll *a*, chlorophyll *b*, and carotenoids.

Treatments of Zinc Sulfate (µmol dm^−3^)	TSS * °Brix		Chlorophyll *a* (mg dm^−1^ DW **)	Chlorophyll *b* (mg dm^−1^ DW **)	Total Carotenoids (mg dm^−1^ DW **)
	Bean	Pod	Leaf		
0.000	0.50 c	5.60 b	0.560 ab	0.056 ab	0.013 a
0.025	6.90 b	6.20 b	0.670 ab	0.067 a	0.013 a
0.050	7.00 b	10.4 a	0.130 b	0.013 a	0.013 a
0.100	7.10 b	6.20 b	0.490 ab	0.049 b	0.013 a
0.200	10.6 a	7.60 ab	0.858 a	0.858 a	0.013 a

* TSS: °Brix; DW: Dry Weight. ** Values with same letters within a column are statistically similar (Tukey; *p* ≤ 0.05). Note: chlorophyll a: results with minus are with letter b.

**Table 5:** Additive formulations established at the experimental site.

Treatments of Iron Sulfate (µmol dm^−3^)	Iron Sulfate (g dm^−3^)	Treatments of Zinc Sulfate (µmol dm^−3^)	Zinc Sulfate (g dm^−3^)
0.000	0	0.000	0
0.025	0.0037977	0.025	0.00404
0.050	0.0075955	0.050	0.00807
0.100	0.0151910	0.100	0.01615
0.200	0.0303820	0.200	0.03229

**Table 6:** Treatments by zinc concentrations.

Zinc Sulfate Concentration (g dm^−3^)	N° Plant			
	1	2	3	4
0	T_1_	T_2_	T_3_	T_4_
0.00404	T_5_	T_6_	T_7_	T_8_
0.00807	T_9_	T_10_	T_11_	T_12_
0.01615	T_13_	T_14_	T_15_	T_16_
0.03229	T_17_	T_18_	T_19_	T_20_

**Table 7:** Treatments by iron concentrations.

Iron Sulfate Concentration (g dm^−3^)	N° Plant			
	1	2	3	4
0	T_1_	T_2_	T_3_	T_4_
0.0037977	T_5_	T_6_	T_7_	T_8_
0.0075955	T_9_	T_10_	T_11_	T_12_
0.0151910	T_13_	T_14_	T_15_	T_16_
0.0303820	T_17_	T_18_	T_19_	T_20_

## Data Availability

Data available on request.
